# Sex-specific pharmacovigilance signals and time-to-onset patterns of drug-related myoclonus and epileptic seizures: a real-world pharmacovigilance analysis

**DOI:** 10.3389/fphar.2026.1907007

**Published:** 2026-08-25

**Authors:** Fu Zhang, Wenjun Yuan, Jingxi Yao, Yushu Wei, Xing Wei, Kexin Sun, Zhenggang Shi, Jing Shang

**Affiliations:** School of Clinical Chinese Medicine, Gansu University of Chinese Medicine, Lanzhou, China

**Keywords:** epileptic seizure, FAERS, myoclonus, pharmacovigilance, reporting odds ratio, time-to-onset

## Abstract

**Background:**

Drug-related myoclonus and epileptic seizures are neurological adverse events that may cause trauma, impaired consciousness, hospitalization, treatment interruption, and life-threatening outcomes. In practice, these events may be difficult to distinguish from neurological diseases, metabolic abnormalities, disease progression, or complications of comorbidities and polypharmacy. Research has focused on individual drugs, case reports, or specific populations, leaving the risk-drug spectrum, sex-related reporting patterns, and onset characteristics insufficiently characterized. Systematic evaluation of these features is important for early identification, optimized monitoring, and individualized risk management.

**Methods:**

FAERS reports from 2004Q1 to 2025Q4 were analyzed. Drug names were standardized using RxNorm, and adverse events were identified using MedDRA 27.1 preferred terms. Primary suspect drugs were evaluated by reporting odds ratio (ROR)-based disproportionality analysis. Sex-stratified analysis, ATC classification, time-to-onset (TTO) analysis, Weibull modeling, and cross-database validation using CVARD were performed.

**Results:**

Reports were concentrated in North America, Europe, and East Asia, with female predominance and most patients aged 18–65 years. A total of 165 myoclonus-related and 235 epileptic seizure-related risk drugs were identified. Nervous system agents predominated, while antiinfectives, antineoplastic and immunomodulating agents, metabolic drugs, and cardiovascular agents also contributed. Bupropion had the highest report count (1,412; 265 myoclonus and 1,147 epileptic seizure reports), followed by tramadol (829). For myoclonus, gabapentin showed strong signals in males (ROR = 23.31, 95% CI: 20.95–25.95) and females (ROR = 18.06, 95% CI: 16.24–20.08), whereas tranexamic acid showed the strongest male signal (ROR = 128.41, 95% CI: 98.29–167.75). For epileptic seizures, bupropion showed signals in males (ROR = 18.08, 95% CI: 16.21–20.17) and females (ROR = 20.21, 95% CI: 18.70–21.85), with tramadol signals in both sexes. Drugs exhibited early-failure Weibull patterns (β < 1). Median TTOs were 16.5 days for pregabalin-related myoclonus and 31.0 days for tramadol-related seizures, but 214.0 days for lamotrigine and 387.0 days for interferon beta-1a. CVARD identified 498 myoclonus-related and 940 seizure-related drugs and reproduced core signals, including clozapine, gabapentin, baclofen, pregabalin, sertraline, bupropion, quetiapine, and olanzapine.

**Conclusion:**

Integrating signal detection, sex-stratified analysis, TTO modeling, and cross-database validation identified core risk drugs and monitoring windows, supporting earlier recognition and individualized management of drug-related neurological adverse events.

## 1 Introduction

Myoclonus and epileptic seizures are clinically important neurological events encountered across diverse patient populations and healthcare settings, contributing to a substantial burden of neurological morbidity and medication-safety concerns. Although their clinical manifestations and diagnostic definitions are not identical, both may involve increased central nervous system excitability, impaired inhibitory regulation, or abnormal synchronization of neural networks (Latorre et al., 2023; Kanner and Bicchi, 2022). They may also share similar precipitating factors, including neurological diseases, metabolic disturbances, systemic illnesses, and medication exposure, and may present with overlapping motor or consciousness-related abnormalities. Myoclonus is characterized by sudden, brief, involuntary muscle contractions or interruptions of muscle tone and may arise from cortical, subcortical, brainstem, or spinal pathways (Latorre et al., 2025a). Epileptic seizures result from abnormal excessive or synchronous neuronal activity in the brain and may manifest as altered consciousness, focal neurological symptoms, or generalized tonic–clonic activity (Riney et al., 2022). Myoclonus may occur in either epileptic or nonepileptic conditions, and myoclonic movements can also form part of certain seizure types. Thus, while the two events should be appropriately distinguished during clinical assessment, their shared mechanisms, overlapping manifestations, and common medication-related triggers support their comparative evaluation within a unified drug-safety framework. Both events may lead to falls, trauma, impaired consciousness, hospitalization, treatment interruption, and potentially life-threatening outcomes (Yamamoto et al., 2025).

The global burden of neurological disorders continues to increase, contributing substantially to disability, mortality, and long-term healthcare needs (Ouyang et al., 2026). Epilepsy is a common chronic neurological disorder characterized by recurrent seizures and considerable physical, psychological, and social consequences (Ioannou et al., 2022). Myoclonus, by contrast, is more often regarded as a neurological symptom, sign, or adverse-event phenotype rather than an independent chronic disease. However, new-onset myoclonus may indicate drug-related neurotoxicity, metabolic disturbance, hypoxic injury, or emerging epileptiform activity (Latorre et al., 2025b). From a medication-safety perspective, jointly evaluating myoclonus and epileptic seizures may therefore provide a more complete picture of drug-related neurological toxicity. Such an approach may identify shared risk drugs, distinguish event-specific drug profiles, and improve recognition of subtle motor abnormalities that could otherwise be overlooked.

Drug-related myoclonus and epileptic seizures are often difficult to recognize and attribute. Both may result from primary neurological diseases, metabolic abnormalities, infections, cerebrovascular lesions, structural brain injury, or medication exposure (Riva et al., 2024). In patients with multisystem comorbidities and polypharmacy, it may be difficult to separate the effects of a suspected drug from disease progression, renal dysfunction, metabolic disturbances, concomitant treatments, and drug–drug interactions (Lucas et al., 2022). Mild limb jerks, tremor-like movements, transient absence-like episodes, fluctuating consciousness, and nonspecific motor abnormalities may also be misinterpreted as manifestations of the underlying disease (Rissardo et al., 2025). These challenges increase the likelihood of delayed recognition and emphasize the need for systematic identification of medications associated with these events.

A broad range of drugs have been reported to induce or aggravate myoclonus and epileptic seizures, including antiepileptic drugs, antipsychotics, antidepressants, opioid analgesics, antibacterials, immunomodulatory agents, and certain cardiovascular and metabolic drugs (Li et al., 2025). Representative drugs include gabapentin, pregabalin, clozapine, quetiapine, venlafaxine, tramadol, and several anti-infective agents (Desai et al., 2019; Gopaul and Altalib, 2024; Nakhaee et al., 2019). Potential mechanisms include reduced GABAergic inhibition, enhanced glutamatergic excitation, altered ion-channel function, dysregulated monoaminergic neurotransmission, drug accumulation, impaired renal elimination, altered blood–brain barrier permeability, and increased central nervous system vulnerability (Wanleenuwat et al., 2020; Janssen et al., 2017). Collectively, these findings suggest that drug-related neurological events arise from interactions among three major domains: drug properties, patient susceptibility, and the clinical medication context. Different combinations of these factors may result in myoclonus, epileptic seizures, or overlapping neurological manifestations.

Sex may further contribute to heterogeneity in drug-related neurological risk. Male and female patients may differ in drug exposure, therapeutic indications, comorbidity profiles, concomitant treatments, and medication-use patterns. These differences may influence the distribution and strength of observed drug–event associations. Sex-stratified evaluation is therefore important because analyses of the overall population may conceal associations that are more prominent in one subgroup or fail to identify signals consistently observed in both sexes. Clarifying these patterns may support more individualized risk recognition and medication monitoring.

Previous evidence has largely been derived from case reports, small clinical series, or analyses focused on a single drug, therapeutic setting, patient population, or neurological outcome (Zou et al., 2025). Consequently, the relationship between the drug-risk spectra of myoclonus and epileptic seizures remains insufficiently characterized. It is not fully understood which drugs are associated with both events, which show relatively event-specific patterns, or whether their therapeutic-class distributions differ. Moreover, sex-related heterogeneity, onset characteristics, and the consistency of major drug associations across different pharmacovigilance systems have rarely been examined within a unified framework (Brabete et al., 2022). Addressing these gaps is important for moving beyond isolated drug–event observations toward a broader and clinically more useful understanding of medication-related neurological toxicity.

Large-scale spontaneous reporting systems offer an opportunity to investigate rare or insufficiently characterized drug-related neurological events in real-world populations. FAERS contains adverse-event reports covering a wide range of drugs and patient groups (Chen et al., 2026), while CVARD provides an independent source for assessing whether major drug-reporting patterns are observed across different pharmacovigilance systems (Xu et al., 2026). In this study, we comparatively evaluated drug-related myoclonus and epileptic seizures using real-world pharmacovigilance data, with particular attention to shared and event-specific drug profiles, therapeutic categories, sex-related patterns, and clinically relevant onset characteristics. By examining two clinically overlapping but conceptually distinct neurological events within a common framework, this study aims to improve recognition of drug-related neurotoxicity, identify clinically important drugs and susceptible populations, and provide evidence for more targeted medication monitoring and earlier intervention.

## 2 Materials and methods

### 2.1 Data source and processing

Adverse event data were obtained from the U.S Food and Drug Administration Adverse Event Reporting System (FAERS). Quarterly ASCII data files covering the period from 2004Q1 to 2025Q4 were downloaded and integrated.

Reports sharing the same CASEID were considered different versions or follow-up reports of the same case. For each CASEID, the record with the latest FDA receipt date (FDA_DT) was retained. When multiple records had the same CASEID and FDA_DT, the record with the highest PRIMARYID was retained. All other FAERS tables were subsequently restricted to the retained PRIMARYIDs.

Drug names were standardized to active-ingredient names using the RxNorm nomenclature system. Brand names, spelling variants, and duplicate records referring to the same standardized drug within an individual case were harmonized and collapsed.

Myoclonus-related and seizure-related events were identified using two prespecified sets of Preferred Terms from the Medical Dictionary for Regulatory Activities version 28.0. The term sets included core clinical phenotypes, subtype-specific events, acute symptomatic seizures, seizure-associated syndromes, and related movement or seizure-like manifestations. The complete lists of PTs are provided in Supplementary Table S1. A case was considered eligible for an outcome when at least one corresponding PT was recorded. Multiple eligible PTs belonging to the same outcome category within one case were counted once at the case–outcome level, and cases meeting both outcome definitions were allowed to contribute to both analyses.

Only drugs designated as primary suspect drugs were included. When a case contained multiple distinct primary suspect drugs, each unique standardized primary-suspect-drug–outcome pair was included once in the drug-specific analysis. Repeated entries of the same standardized drug within the same case were collapsed. Demographic and clinical characteristics were summarized according to sex, age, body weight, indication, reporting country, and clinical outcome.

### 2.2 Signal detection algorithm

Disproportionality analyses were performed separately for myoclonus-related and seizure-related events. For each standardized primary-suspect-drug–outcome pair, a two-by-two contingency table was constructed using the remaining FAERS reports as the comparator. Each case was counted no more than once for a given standardized drug–outcome pair.

The reporting odds ratio (ROR) was used as the primary signal-detection measure because it is widely applied in case–noncase pharmacovigilance analyses and provides an interpretable estimate with a corresponding confidence interval. A positive ROR signal was defined as at least four reports for the drug–outcome pair and a lower limit of the 95% confidence interval greater than 1 (Wang et al., 2026).

To evaluate signal stability, the proportional reporting ratio (PRR) and Bayesian information component (IC) were additionally calculated as complementary analyses. A PRR signal was considered positive when the number of reports was at least three, the PRR was ≥2, and the chi-square value was ≥4. An IC signal was considered positive when the lower limit of its 95% credibility interval (IC025) was greater than 0. Signals detected consistently by the ROR and at least one complementary method were considered more robust. The definitions and calculation formulas are provided in Supplementary Material 2.

Sex-stratified analyses were conducted separately among reports with known male and female sex. Reports with missing or unknown sex were excluded from the stratified analyses. All data processing and statistical analyses were performed using R version 4.4.0.

### 2.3 Time-to-onset analysis

To further characterize the temporal patterns of myoclonus-related and seizure-related events, a time-to-onset (TTO) analysis was performed (Pacaci et al., 2026). TTO was calculated separately for each eligible primary-suspect-drug–outcome pair as the interval, in days, between the corresponding drug initiation date and the adverse event onset date. Reports were directly excluded from the TTO analysis if the drug initiation date or adverse event onset date was missing, if the calculated TTO was less than or equal to 0 days, or if the recorded dates were chronologically inconsistent or otherwise invalid. No missing date information was imputed.

After applying these exclusion criteria, drugs were ranked according to the number of reports with valid and calculable TTO information. For each outcome, the three drugs with the largest numbers of eligible TTO records were selected to ensure sufficient data for describing the onset-time distribution and fitting the Weibull model. Accordingly, GABAPENTIN, LAMOTRIGINE, and PREGABALIN were selected for myoclonus-related events, whereas BUPROPION, TRAMADOL, and INTERFERON BETA 1 A were selected for seizure-related events.

The median and interquartile range (IQR) were used to describe the central tendency and dispersion of TTO, and violin plots were generated to visualize differences in TTO distributions among drugs. Weibull distribution models were fitted to estimate the shape parameter β and scale parameter α. A β value less than 1 indicated an early-failure pattern, suggesting that adverse events were more frequently reported during the early phase after treatment initiation. A β value approximately equal to 1 indicated a random-failure pattern, whereas a β value greater than 1 indicated a wear-out pattern, suggesting an increasing reporting tendency with prolonged exposure. All TTO analyses and graphical visualizations were performed using R version 4.4.0.

### 2.4 Cross-database consistency assessment using CVARD

The Canada Vigilance Adverse Reaction Database (CVARD) was used as an independent data source to assess the cross-database consistency of major drug-reporting patterns identified in FAERS. As a spontaneous adverse reaction reporting database independent of FAERS, CVARD can be used to assess the consistency of major risk-drug signals across different pharmacovigilance systems.

Using the same adverse event definitions as those applied in the FAERS analysis, adverse event reports related to myoclonus and seizure-related events were separately extracted from CVARD. Drug names were uniformly formatted, including removal of leading and trailing spaces, standardization of letter case, and, where possible, merging of generic names, brand names, and different spelling variants of the same drug. Subsequently, the number of drug-related reports for myoclonus and seizure-related events in CVARD was calculated and compared with the risk drugs identified in FAERS.

The cross-database consistency assessment consisted mainly of two parts. First, for the top 20 risk drugs associated with myoclonus and seizure-related events in FAERS, the corresponding number of reports in CVARD was calculated, and the combined number of reports from the two databases was obtained as ALL = FAERS + CVARD. The reporting distribution of core risk drugs across the two databases was visualized using heatmaps. Because the number of reports differed substantially between FAERS and CVARD, a logarithmic scale was applied in the heatmaps to improve visualization of drugs with lower reporting frequencies.

**FIGURE 1:**
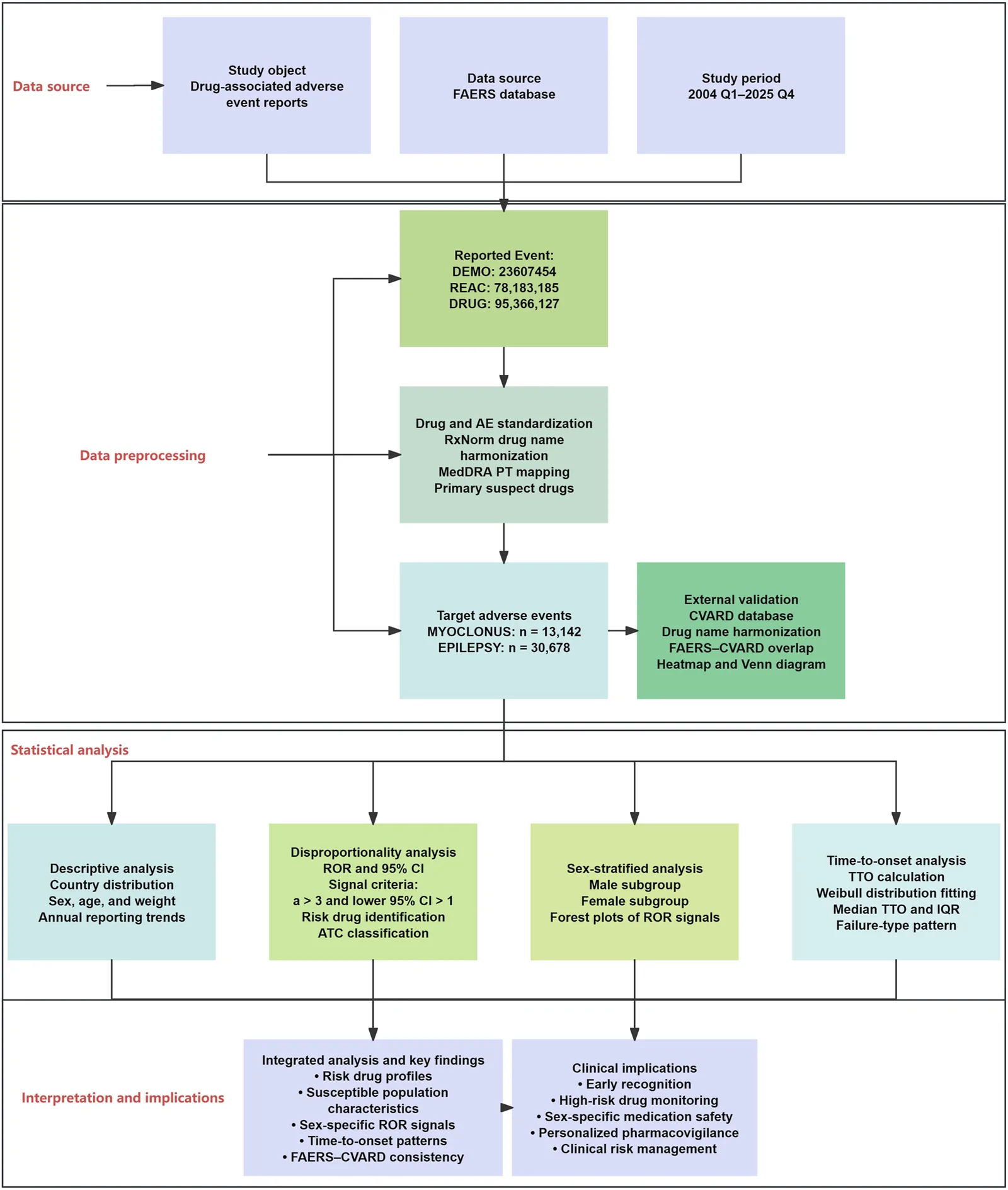
Analytical workflow for adverse event reports related to drug-associated myoclonus and seizure-related events.

Second, risk-drug lists for myoclonus and seizure-related events were constructed separately for FAERS and CVARD. Area-proportional Venn diagrams were used to show the numbers of database-specific and overlapping drugs. Drugs shared by both databases were considered to have better external consistency, whereas drugs appearing in only one database may indicate signal heterogeneity attributable to regional differences, reporting systems, or medication-use patterns. The data extraction and analysis workflow is shown in Figure 1.

## 3 Results

### 3.1 Demographic characteristics of adverse event reports related to drug-associated myoclonus and seizure-related events

**FIGURE 2:**
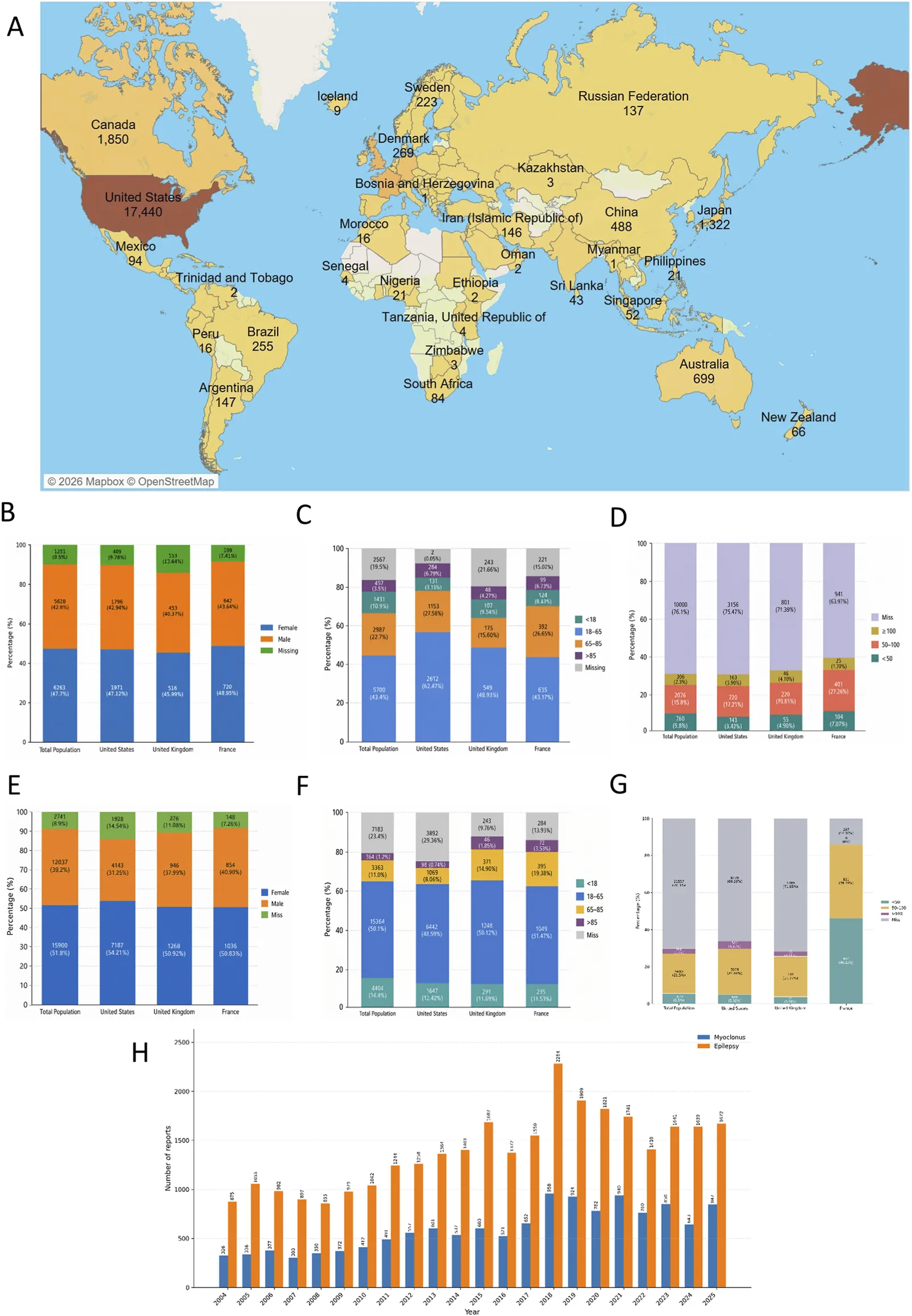
Geographic distribution, demographic characteristics, and annual reporting trends of adverse events related to drug-associated myoclonus and seizure-related events in the FAERS database. **(A)** Global geographic distribution of adverse event reports related to drug-associated myoclonus and seizure-related events after aggregation by country or region. Color intensity indicates the number of reports in each country or region. **(B–D)** Sex, age, and body-weight distributions of myoclonus-related reports in the overall population and in the United States, the United Kingdom, and France, which were countries with relatively high reporting numbers. **(E–G)** Sex, age, and body-weight distributions of epileptic seizure-related reports in the overall population and in the United States, the United Kingdom, and France. **(H)** Annual reporting trends of adverse events related to drug-associated myoclonus and seizure-related events from 2004 to 2025. Stratified bar charts show the proportions of each subgroup, with values presented as report numbers and percentages. Missing data were retained as independent categories. The geographic distribution reflects the distribution of reporting sources in the FAERS database and does not represent true incidence differences across countries or regions.

Figure 2A shows the global geographic distribution of adverse event reports related to drug-associated myoclonus and seizure-related events in the FAERS database. The reports covered multiple countries and regions; however, their distribution was uneven and was mainly concentrated in North America, Europe, and East Asia. The United States had the highest number of reports, with 17,440 cases, substantially exceeding those from other countries. This was followed by the United Kingdom, France, Germany, Canada, and Japan. Based on the relatively high number of reports and their representativeness, the United States, the United Kingdom, and France were further selected and compared with the overall reporting population to analyze sex, age, and body-weight distributions across different regions.

Among myoclonus-related adverse event reports, the proportion of female reports was slightly higher than that of male reports. In the overall population, there were 6,263 female reports, accounting for 47.7%, and 5,628 male reports, accounting for 42.8%; sex was missing in 1,251 reports, accounting for 9.5%. In the United States, the United Kingdom, and France, the proportions of female reports were 47.12%, 45.99%, and 48.95%, respectively, all of which were slightly higher than or close to the corresponding proportions of male reports (Figure 2B). Regarding age distribution, myoclonus reports were mainly concentrated in the 18–65-year age group, which accounted for 43.4% of the overall population. This was followed by the 65–85-year age group, accounting for 22.7%. The proportions of patients aged <18 years and >85 years were 10.9% and 3.5%, respectively. The United States had the highest proportion of reports in the 18–65-year age group, at 62.47%, whereas the corresponding proportions in the United Kingdom and France were 48.93% and 43.17%, respectively (Figure 2C). For body-weight distribution, the proportion of missing body-weight information was high among myoclonus reports, with an overall missing rate of 76.1%. Among reports with available body-weight information, the 50–100 kg group accounted for the largest proportion, at 15.8%, whereas the <50 kg and ≥100 kg groups accounted for 5.8% and 2.3%, respectively. A high proportion of missing body-weight information was also observed in the United States, the United Kingdom, and France, indicating that body-weight-related findings should be interpreted with caution (Figure 2D).

Among epileptic seizure-related adverse event reports, the proportion of female reports was also higher than that of male reports. In the overall population, there were 15,900 female reports, accounting for 51.8%, and 12,037 male reports, accounting for 39.2%; sex was missing in 2,741 reports, accounting for 8.9%. The proportions of female reports in the United States, the United Kingdom, and France were 54.21%, 50.92%, and 50.83%, respectively, all of which were higher than the corresponding proportions of male reports (Figure 2E). Regarding age distribution, epileptic seizure reports were also dominated by the 18–65-year age group, which accounted for 50.1% of the overall population. The proportions of patients aged <18 years, 65–85 years, and >85 years were 14.4%, 11.0%, and 1.2%, respectively, and age was missing in 23.4% of reports. Age structure differed to some extent across countries. In the United States, the 18–65-year age group accounted for 48.59%, with a relatively high proportion of missing age information. In France, the 65–85-year age group accounted for 19.38%, which was higher than the overall level (Figure 2F). For body-weight distribution, missing body-weight information was also frequent among epileptic seizure reports, with an overall missing rate of 70.3%. Among reports with available body-weight information, the 50–100 kg group accounted for 21.5%, whereas the <50 kg and ≥100 kg groups accounted for 5.5% and 2.7%, respectively. Notably, the proportion of missing body-weight information was relatively lower in reports from France, while the proportions of the <50 kg and 50–100 kg groups were relatively higher, at 46.12% and 39.79%, respectively (Figure 2G).

Annual trend analysis showed that the number of adverse event reports related to drug-associated myoclonus and seizure-related events generally increased from 2004 to 2025, with fluctuations across years. Both types of events reached their peak in 2018, with 958 reports of myoclonus and 2,284 reports of seizure-related events. After 2018, the number of reports for both adverse events fluctuated but remained at relatively high levels overall. In each year, the number of epileptic seizure reports was higher than that of myoclonus reports, suggesting a greater reporting burden of epileptic seizure-related adverse events in the FAERS database (Figure 2H).

Overall, reports of drug-associated myoclonus and seizure-related events showed clear geographic concentration and certain similarities in demographic characteristics, including a slightly higher proportion of female reports, predominance of the 18–65-year age group, and a high proportion of missing body-weight information. It should be noted that these results reflect reporting characteristics and the distribution of reporting sources in the FAERS database, rather than true differences in incidence across countries, sexes, age groups, or body-weight categories.

### 3.2 Risk drugs associated with drug-related myoclonus and seizure-related events

**FIGURE 3:**
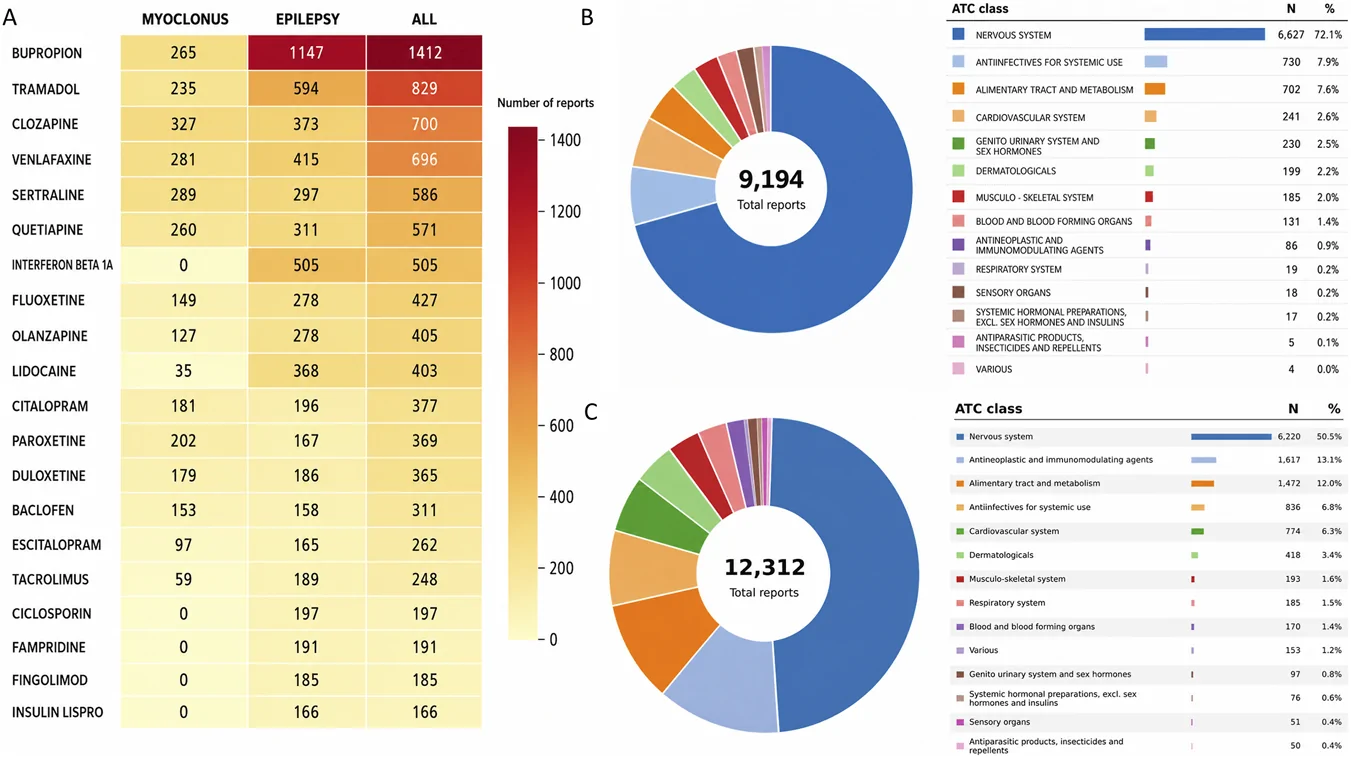
Risk drugs associated with drug-related myoclonus and seizure-related events and their distribution by ATC classification. **(A)** Heatmap of the top 20 drugs with the highest numbers of reports for risk signals related to drug-associated myoclonus and seizure-related events, ranked in descending order by total number of reports. Columns represent the number of myoclonus reports, epileptic seizure reports, and their combined total, respectively. **(B)** Distribution of ATC categories for drugs associated with myoclonus. **(C)** Distribution of ATC categories for drugs associated with seizure-related events. In the circular plots, arc length represents the relative number of reports in each ATC category, and values are presented as report counts and percentages.

Using disproportionality analysis, we identified a total of 165 risk drugs associated with myoclonus and 235 risk drugs associated with seizure-related events. The top 20 drugs ranked by the number of epileptic seizure reports are shown in Figure 3A. Overall, most of the top 20 drugs were associated with both myoclonus and seizure-related events. BUPROPION had the highest total number of reports, with 1,412 cases, including 265 reports of myoclonus and 1,147 reports of seizure-related events. This was followed by TRAMADOL, with a total of 829 reports. Several psychotropic and neurological agents, including CLOZAPINE, VENLAFAXINE, SERTRALINE, and QUETIAPINE, also ranked prominently. In contrast, some drugs were mainly associated with epileptic seizure reports. For example, INTERFERON BETA 1A, CICLOSPORIN, FAMPRIDINE, FINGOLIMOD, and INSULIN LISPRO were not identified in association with myoclonus in the present study, but all had reports related to seizure-related events.

The identified risk drugs were further classified according to the Anatomical Therapeutic Chemical (ATC) system. Drugs associated with myoclonus were predominantly concentrated in the nervous system category, with 6,627 reports, accounting for 72.1% of all myoclonus-related drug reports. This was followed by antiinfectives for systemic use, with 730 reports (7.9%), and alimentary tract and metabolism drugs, with 702 reports (7.6%). Other categories included cardiovascular system drugs, genitourinary system and sex hormones, dermatologicals, and musculoskeletal system drugs, although the number of reports in these categories was relatively small (Figure 3B). Similarly, drugs associated with seizure-related events were mainly distributed in the nervous system category, with 6,220 reports. In addition, antineoplastic and immunomodulating agents, alimentary tract and metabolism drugs, antiinfectives for systemic use, and cardiovascular system drugs also accounted for substantial proportions (Figure 3C).

These findings indicate that drug-related myoclonus and seizure-related events are both primarily associated with nervous system medications. However, the ATC distribution of drugs associated with seizure-related events was broader, involving multiple therapeutic areas, including antineoplastic and immunomodulating agents, metabolic drugs, antiinfectives, and cardiovascular agents.

### 3.3 Sex-stratified analysis

**FIGURE 4:**
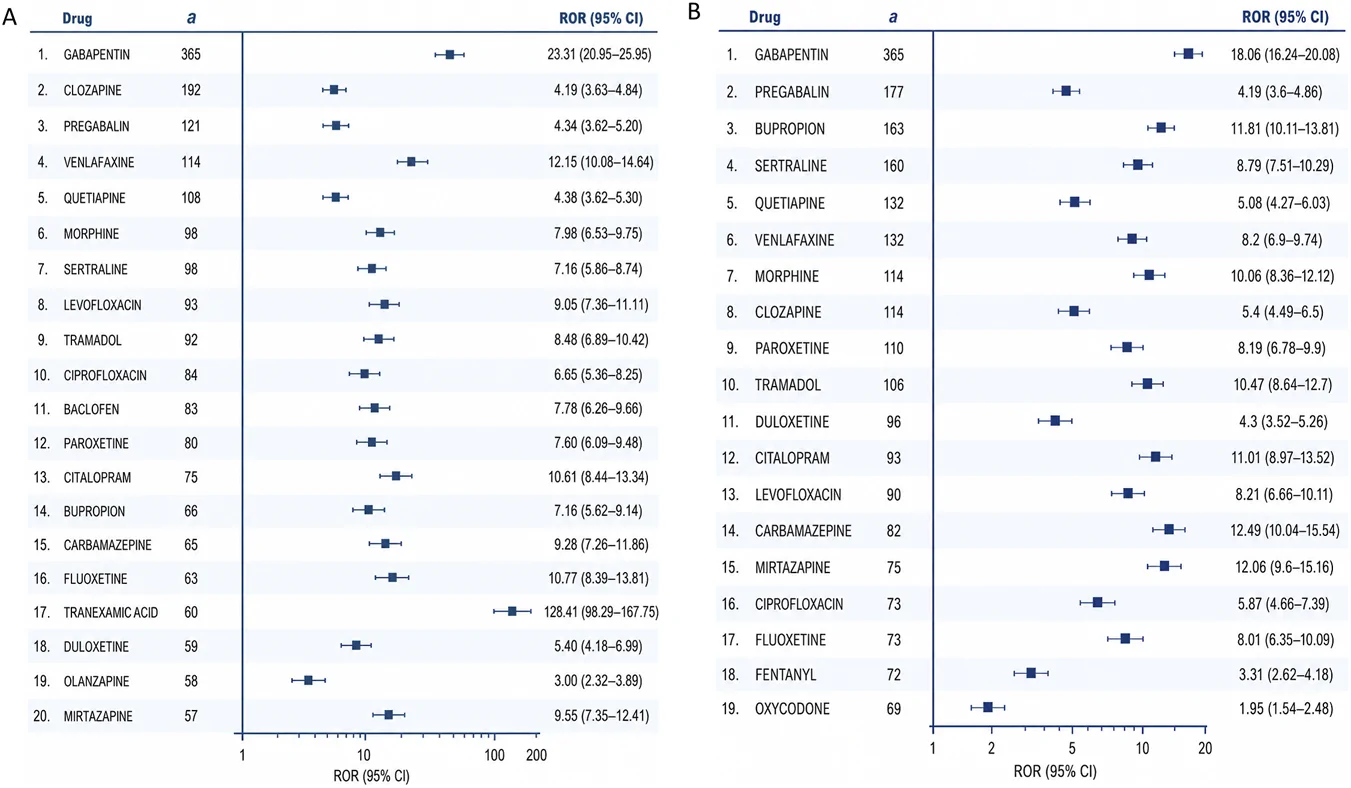
Sex-stratified disproportionality analysis of risk drugs associated with drug-related myoclonus adverse events. **(A)** Forest plot of the top 20 drugs ranked by the number of myoclonus adverse event reports in the male population. **(B)** Forest plot of the leading drugs ranked by the number of myoclonus adverse event reports in the female population. The number of myoclonus reports for each drug is denoted as a. Squares represent reporting odds ratios (RORs), and horizontal lines represent 95% confidence intervals (95% CIs). The vertical reference line indicates ROR = 1. An ROR >1 with a 95% CI that does not cross 1 indicates a positive disproportionality signal. ROR values are displayed on a logarithmic scale.

To characterize sex-stratified reporting patterns of myoclonus-related adverse events, disproportionality analyses were performed separately in male and female reports for the leading signal-associated drugs. Forest plots present the RORs and corresponding 95% confidence intervals (CIs), with detailed drug-specific estimates shown in Figure 4.

GABAPENTIN was the most frequently reported drug in both male and female populations and showed strong disproportionality signals in males [365 reports; ROR = 23.31 (95% CI: 20.95–25.95)] and females [365 reports; ROR = 18.06 (95% CI: 16.24–20.08)]. Several drugs, including PREGABALIN, QUETIAPINE, VENLAFAXINE, SERTRALINE, CITALOPRAM, CARBAMAZEPINE, and FLUOXETINE, showed positive myoclonus-related signals in both sex subgroups. Among male reports, TRANEXAMIC ACID showed the highest ROR [60 reports; ROR = 128.41 (95% CI: 98.29–167.75)], whereas BUPROPION, MIRTAZAPINE, and MORPHINE were among the notable signals identified in female reports.

Although numerical differences in ROR estimates were observed between male and female subgroups, these estimates were not subjected to formal between-sex interaction testing. In addition, after reports with missing or unknown sex were excluded, some drug-specific strata contained limited numbers of reports, which reduced the reliability of formal comparisons. Therefore, these findings should be interpreted as exploratory sex-stratified reporting patterns rather than statistically confirmed sex-specific effects.

**FIGURE 5:**
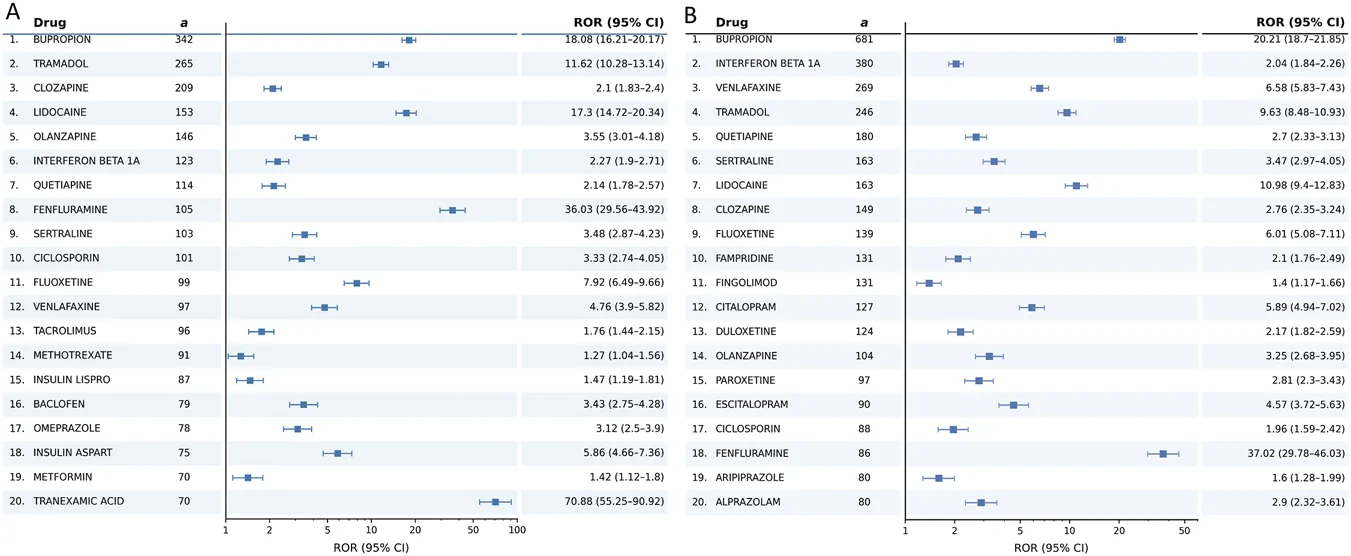
Sex-stratified disproportionality analysis of risk drugs associated with drug-related epileptic seizure adverse events. **(A)** Forest plot of the top 20 drugs ranked by the number of epileptic seizure adverse event reports in the male population. **(B)** Forest plot of the top 20 drugs ranked by the number of epileptic seizure adverse event reports in the female population. The number of epileptic seizure reports for each drug is denoted as a. Squares represent reporting odds ratios (RORs), and horizontal lines represent 95% confidence intervals (95% CIs). The vertical reference line indicates ROR = 1. An ROR >1 with a 95% CI that does not cross 1 indicates a positive disproportionality signal. ROR values are displayed on a logarithmic scale.

Among epileptic seizure-related adverse events, the risk-drug spectra in the male and female populations also showed both overlap and sex-specific differences (Figure 5). In the male population, BUPROPION had the highest number of reports, with 342 cases and an ROR of 18.08 (95% CI: 16.21–20.17) (Figure 5A). This was followed by TRAMADOL [265 reports; ROR = 11.62 (95% CI: 10.28–13.14)], CLOZAPINE [209 reports; ROR = 2.10 (95% CI: 1.83–2.40)], LIDOCAINE [153 reports; ROR = 17.30 (95% CI: 14.72–20.34)], and OLANZAPINE [146 reports; ROR = 3.55 (95% CI: 3.01–4.18)]. Although TRANEXAMIC ACID had only 70 reports, it showed the strongest signal, with an ROR of 70.88 (95% CI: 55.25–90.92). FENFLURAMINE also exhibited a strong signal, with an ROR of 36.03 (95% CI: 29.56–43.92). In addition, FLUOXETINE, INSULIN ASPART, VENLAFAXINE, and BACLOFEN showed RORs of 7.92, 5.86, 4.76, and 3.43, respectively.

In the female population, BUPROPION was likewise the most frequently reported drug, with 681 reports and an ROR of 20.21 (95% CI: 18.70–21.85) (Figure 5B). INTERFERON BETA 1 A had 380 reports, with an ROR of 2.04 (95% CI: 1.84–2.26); VENLAFAXINE had 269 reports, with an ROR of 6.58 (95% CI: 5.83–7.43); TRAMADOL had 246 reports, with an ROR of 9.63 (95% CI: 8.48–10.93); and QUETIAPINE had 180 reports, with an ROR of 2.70 (95% CI: 2.33–3.13). The strongest signal in the female population was observed for FENFLURAMINE, with an ROR of 37.02 (95% CI: 29.78–46.03), followed by BUPROPION and LIDOCAINE [ROR = 10.98 (95% CI: 9.40–12.83)]. In addition, FLUOXETINE, CITALOPRAM, ESCITALOPRAM, and ALPRAZOLAM showed clear risk signals, with RORs of 6.01, 5.89, 4.57, and 2.90, respectively.

Overall, BUPROPION, TRAMADOL, VENLAFAXINE, QUETIAPINE, SERTRALINE, LIDOCAINE, CLOZAPINE, FLUOXETINE, OLANZAPINE, CITALOPRAM, DULOXETINE, and BACLOFEN showed epileptic seizure-related signals in both male and female populations. TRANEXAMIC ACID showed a stronger signal in males, whereas INTERFERON BETA 1A, FAMPRIDINE, FINGOLIMOD, ESCITALOPRAM, and ALPRAZOLAM appeared mainly among the top 20 risk drugs in females.

### 3.4 Time-to-onset analysis

**FIGURE 6:**
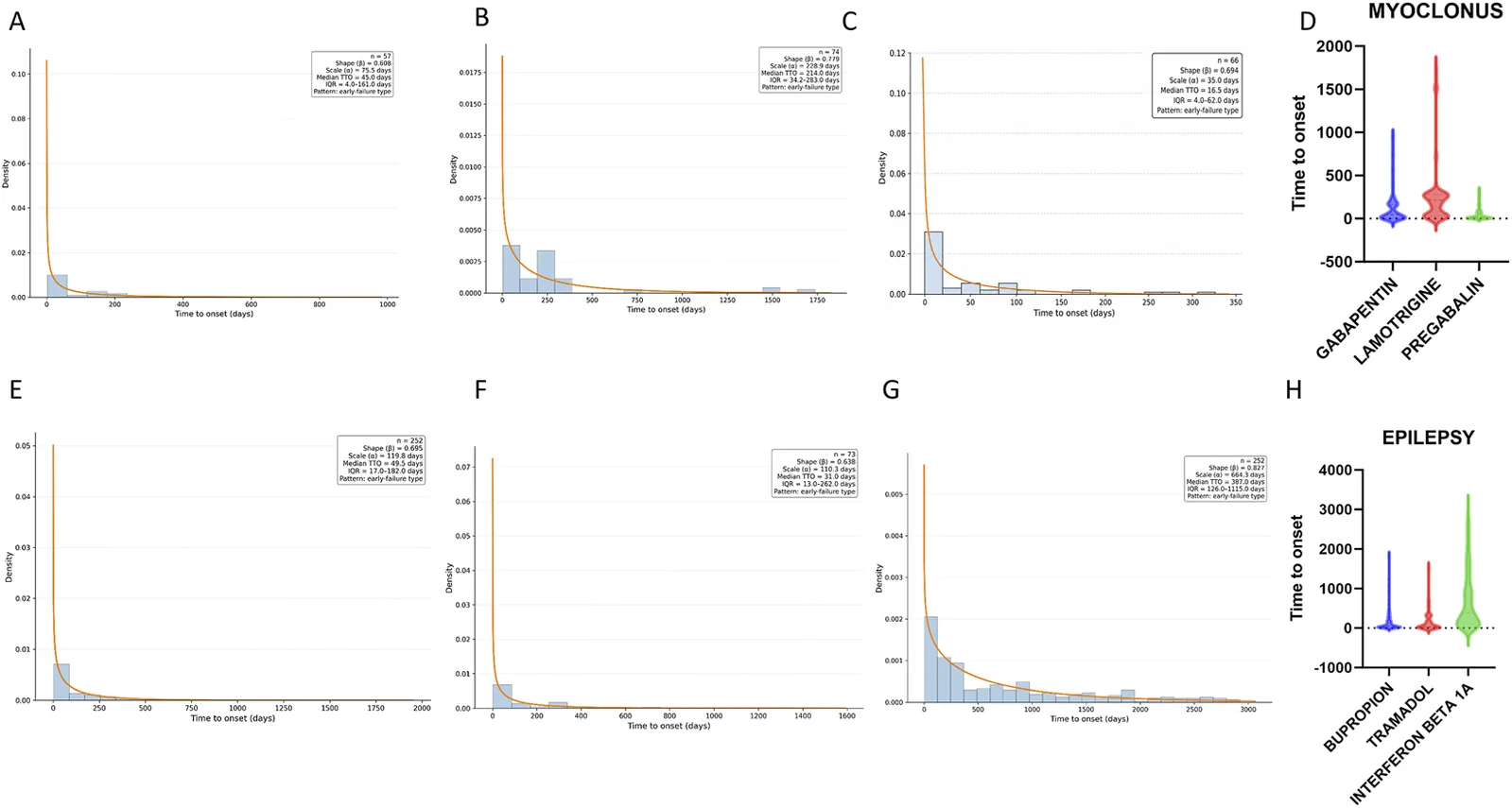
Time-to-onset distribution characteristics of representative risk drugs associated with myoclonus and epileptic seizure adverse events. **(A–C)** Weibull distribution fitting curves for the time-to-onset (TTO) of myoclonus-related adverse event reports associated with GABAPENTIN, LAMOTRIGINE, and PREGABALIN, respectively. **(D)** Violin plot comparing the TTO distributions of these three myoclonus-related risk drugs. **(E–G)** Weibull distribution fitting curves for the TTO of epileptic seizure-related adverse event reports associated with BUPROPION, TRAMADOL, and INTERFERON BETA 1A, respectively. **(H)** Violin plot comparing the TTO distributions of these three epileptic seizure-related risk drugs. Histograms represent the observed TTO distributions, and the fitted curves represent the Weibull distribution results. The shape parameter β, scale parameter α, median TTO, interquartile range (IQR), and failure type are provided for each drug. A β value < 1 indicates an early-failure distribution, suggesting that the related adverse events are more likely to occur during the early phase after treatment initiation.

After applying the date-related exclusion criteria, the three drugs with the largest numbers of valid and calculable TTO records for each outcome were selected for analysis. Their TTO distributions and Weibull model parameters are presented in Figure 6.

For myoclonus-related adverse events, the median TTO ranged from 16.5 days for PREGABALIN [IQR: 4.0–62.0 days; β = 0.694] to 214.0 days for LAMOTRIGINE [IQR: 34.3–283.0 days; β = 0.779], while GABAPENTIN showed an intermediate median TTO of 45.0 days [IQR: 4.0–161.0 days; β = 0.608]. All three β values were below 1, indicating early-failure reporting patterns. However, LAMOTRIGINE showed a later and more widely distributed onset pattern than GABAPENTIN and PREGABALIN (Figures 6A–D).

For seizure-related adverse events, the median TTO was 31.0 days for TRAMADOL [IQR: 13.0–262.0 days; β = 0.638] and 49.5 days for BUPROPION [IQR: 17.0–182.0 days; β = 0.695]. INTERFERON BETA 1 A showed a substantially longer median TTO of 387.0 days [IQR: 126.0–1,115.0 days; β = 0.827]. Although all three drugs also exhibited β values below 1, the onset distribution for INTERFERON BETA 1 A was markedly later and more dispersed (Figures 6E–H).

Overall, the representative drugs predominantly exhibited early-failure reporting patterns, supporting close monitoring during the early phase after treatment initiation. Nevertheless, the considerable variation in median TTO indicates that monitoring periods should also account for drug-specific onset characteristics, particularly for drugs with delayed and widely distributed events.

### 3.5 Cross-database consistency assessment using the canadian database

The Canada Vigilance Adverse Reaction Database (CVARD) was used as an independent data source to assess the cross-database consistency of drug-reporting patterns identified in FAERS. In CVARD, eligible reports involved 498 drugs for myoclonus-related events and 940 drugs for seizure-related events.

**FIGURE 7:**
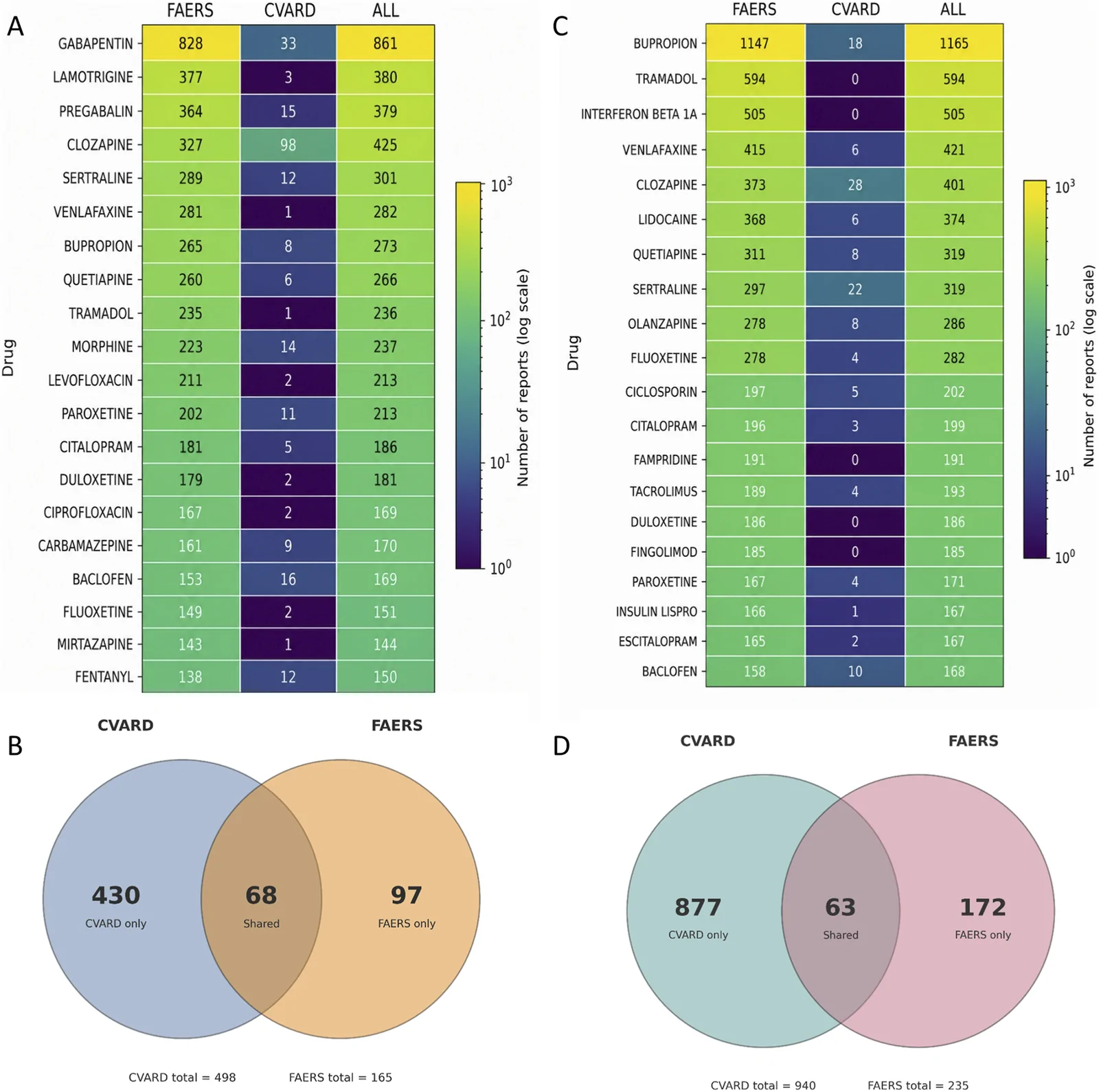
Cross-database consistency assessment of risk signals for drug-related myoclonus and seizure-related events using the Canadian CVARD database. **(A)** Heatmap showing the number of reports for the top 20 myoclonus-related risk drugs identified in FAERS in both the FAERS and CVARD databases. **(B)** Area-proportional Venn diagram showing the overlap of myoclonus-related risk drugs between FAERS and CVARD. **(C)** Heatmap showing the number of reports for the top 20 epileptic seizure-related risk drugs identified in FAERS in both the FAERS and CVARD databases. **(D)** Area-proportional Venn diagram showing the overlap of epileptic seizure-related risk drugs between FAERS and CVARD. In the heatmaps, FAERS and CVARD represent the number of reports in the two independent pharmacovigilance databases, and “ALL” represents the combined number of reports from both databases. Color intensity indicates the number of reports, which is displayed on a logarithmic scale. The Venn diagrams show the numbers of database-specific risk drugs and risk drugs shared by both databases.

For myoclonus-related events, all of the top 20 signal-associated drugs identified in FAERS were also represented in CVARD (Figure 7A). CLOZAPINE, GABAPENTIN, BACLOFEN, PREGABALIN, MORPHINE, FENTANYL, SERTRALINE, and PAROXETINE were among the more frequently reported drugs in CVARD. Overall, 68 drugs were reported in both databases, whereas 97 were specific to FAERS and 430 were specific to CVARD (Figure 7B).

For seizure-related events, most of the top 20 signal-associated drugs identified in FAERS were also reported in CVARD (Figure 7C). CLOZAPINE, SERTRALINE, BUPROPION, BACLOFEN, QUETIAPINE, and OLANZAPINE showed relatively higher report counts in CVARD. However, no corresponding CVARD reports were identified for several leading FAERS drugs, including TRAMADOL, INTERFERON BETA 1A, FAMPRIDINE, DULOXETINE, and FINGOLIMOD. A total of 63 drugs were shared between the two databases, whereas 172 were specific to FAERS and 877 were specific to CVARD (Figure 7D).

Overall, several drugs, including CLOZAPINE, BUPROPION, SERTRALINE, QUETIAPINE, VENLAFAXINE, and BACLOFEN, showed consistent reporting across the two databases. Nevertheless, substantial database-specific differences were also observed. These findings indicate cross-database reporting consistency for selected drugs rather than independent confirmation of causal associations. Differences between FAERS and CVARD may reflect variation in geographic coverage, prescribing practices, drug availability, population characteristics, reporting behavior, coding procedures, and database structure. Database-specific findings should therefore be interpreted cautiously because sparse reporting and reporting bias may contribute to unstable or false-positive associations.

## 4 Discussion

In this study, we systematically characterized the real-world reporting patterns of myoclonus-related and seizure-related adverse events using FAERS and further assessed the cross-database consistency of major drug-reporting patterns using CVARD. The two outcomes showed both shared and distinct patterns in demographic characteristics, therapeutic categories, sex-stratified reporting, and time to onset. Nervous system agents were most frequently represented, although antiinfectives, antineoplastic and immunomodulating agents, alimentary tract and metabolism drugs, and cardiovascular drugs were also involved. The selected signal-associated drugs generally showed Weibull shape parameters below 1, indicating that their reports tended to occur relatively early after treatment initiation. Several drug-reporting patterns were also observed in CVARD, supporting cross-database consistency for selected findings.

The findings should be interpreted within the context of spontaneous-reporting pharmacovigilance. Disproportionality measures identify drug–event combinations reported more frequently than expected relative to other reports in the database; they do not establish causality or quantify the incidence, absolute risk, or relative risk of an adverse event. Signal magnitude may also be influenced by reporting volume, drug utilization, therapeutic indications, co-medications, disease severity, and reporting behavior. Accordingly, the identified associations should be regarded as hypothesis-generating safety signals that support further clinical evaluation rather than as confirmed drug-induced effects.

The significance of this study lies in the comparative evaluation of myoclonus and seizure-related events within the same pharmacovigilance framework, which may help identify shared and event-specific drug-reporting patterns for these two neurological adverse events. Myoclonus often presents as brief limb jerks, altered muscle tone, or nonspecific abnormal movements, and is more likely than seizure-related events to be overlooked or misinterpreted as a manifestation of the underlying disease in clinical practice (Zimmern and Minassian, 2024). Therefore, pharmacovigilance monitoring should not focus only on typical seizure-related events, but should also pay attention to early warning signs such as new-onset myoclonus, transient absence-like episodes, fluctuating consciousness, and focal abnormal movements (Zhou et al., 2022).

### 4.1 Demographic reporting patterns and clinical considerations

This study showed that reports of drug-related myoclonus and seizure-related events were mainly submitted from countries such as the United States, the United Kingdom, France, Germany, Canada, and Japan. This geographic concentration is more likely related to the scale of medication use, maturity of reporting systems, reporting awareness, and database coverage, rather than directly reflecting true differences in risk. Notably, demographic characteristics also varied across countries. For example, among myoclonus reports from the United States, individuals aged 18–65 years accounted for a relatively high proportion, suggesting that these reports may have mainly originated from young, middle-aged, and older adult medication users. The age structures in the United Kingdom and France differed to some extent. In particular, among epileptic seizure reports from France, the proportion of individuals aged 65–85 years was higher than the overall level, indicating a relatively greater representation of older adults in reports from certain regions. In addition, the proportion of female reports for myoclonus was slightly lower in the United Kingdom than in the United States and France, indicating that sex and age distributions across countries may be influenced by disease spectrum, medication-use patterns, healthcare accessibility, and reporting behavior (Holm et al., 2017). Therefore, the geographic distribution not only reflects differences in reporting sources, but also indicates that population structure and medication background should be considered when interpreting drug-related neurological adverse events across regions.

From the perspective of overall demographic characteristics, both events were slightly more frequently reported in females and were mainly concentrated in the 18–65-year age group. This may be related to greater opportunities for drug exposure in this age range, higher treatment demands for psychiatric and neurological disorders, increased chronic disease management, and more frequent polypharmacy (Osanlou et al., 2022). Meanwhile, elderly patients often have reduced renal function, altered hepatic drug metabolism, hypoalbuminemia, changes in blood–brain barrier permeability, multiple comorbidities, and concomitant medication use. These factors may increase central nervous system drug exposure and lower the threshold for myoclonus or seizure-related events (Gronich, 2024). For elderly patients receiving gabapentin, pregabalin, opioids, antiinfective agents, or immunomodulatory drugs, drug-related causes should be promptly considered when new-onset limb jerks, fluctuating consciousness, falls, or transient absence-like episodes occur (Coluzzi et al., 2020).

In terms of drug categories, myoclonus-related drugs were mainly concentrated among nervous system agents, followed by antiinfectives and alimentary tract and metabolism drugs. Epileptic seizure-related drugs were also predominantly nervous system agents, but antineoplastic and immunomodulating agents, antiinfectives, metabolic drugs, and cardiovascular drugs were also prominent. These findings suggest that drug-related myoclonus and seizure-related events are not limited to neurological or psychiatric medications. Potential adverse reactions related to increased neural excitability should also be considered during the treatment of infections, tumors, autoimmune diseases, metabolic disorders, and perioperative conditions.

### 4.2 Sex differences and risk-drug profiles

Sex-stratified analysis showed that the risk-drug profiles for both events overlapped between males and females, while also exhibiting certain differences. For myoclonus, GABAPENTIN was the most frequently reported drug in both males and females and showed a strong signal. PREGABALIN, QUETIAPINE, VENLAFAXINE, SERTRALINE, CITALOPRAM, CARBAMAZEPINE, and FLUOXETINE also appeared repeatedly across sex subgroups, suggesting that psychotropic and neurological agents remain important sources of drug-related myoclonus (Janssen et al., 2017).

Mechanistically, gabapentin and pregabalin primarily act on the α2δ subunit of voltage-gated calcium channels. Although they are commonly used for neuropathic pain and as adjunctive therapy for epilepsy, they may still induce myoclonus or other abnormal movements in the presence of renal impairment, drug accumulation, rapid dose escalation, or increased central nervous system susceptibility (Chincholkar, 2020). Myoclonus associated with antipsychotics and antidepressants may be related to dysregulation of dopaminergic, serotonergic, noradrenergic, and GABAergic neurotransmission (Spigset et al., 1997). Clinically, if new-onset myoclonus occurs after the use of clozapine, quetiapine, venlafaxine, fluoxetine, paroxetine, citalopram, or related agents, particularly in the context of dose adjustment, declining renal function, or changes in concomitant medications, drug-related myoclonus should be considered in the differential diagnosis.

For seizure-related events, BUPROPION was the most frequently reported drug in both males and females and showed a strong signal, consistent with its known potential to lower the seizure threshold. The risk associated with BUPROPION may be influenced by dose, formulation type, rapid dose escalation, eating disorders, withdrawal from alcohol or sedative drugs, a history of epilepsy, and concomitant use of other drugs that lower the seizure threshold (Alberter et al., 2026). TRAMADOL also showed clear epileptic seizure signals in both sexes. Its mechanism may involve μ-opioid receptor activity, inhibition of monoamine reuptake, and reduced GABAergic inhibition (Lagard et al., 2022). Particular attention should be paid to tramadol-related seizure risk in patients receiving concomitant antidepressants or antipsychotics, or in those with a history of epilepsy.

Notably, TRANEXAMIC ACID showed high signal strength in both myoclonus and epileptic seizure reports among males. It may increase central nervous system excitability by inhibiting GABA_A receptor- and glycine receptor-mediated inhibitory neurotransmission (Lecker et al., 2016). This finding suggests that postoperative neurological monitoring should be strengthened in patients receiving high-dose tranexamic acid during the perioperative period, particularly those with renal insufficiency or a history of neurological disease (Liu et al., 2024). However, a high ROR does not equate to a high incidence rate and should therefore be interpreted cautiously in relation to the specific clinical context of drug use.

Some drugs were more prominent in female epileptic seizure reports, including INTERFERON BETA 1A, FAMPRIDINE, FINGOLIMOD, ESCITALOPRAM, and ALPRAZOLAM. This pattern may be related to sex differences in indication distribution, exposure base, pharmacokinetic characteristics, or reporting behavior (Zucker and Prendergast, 2020). For example, multiple sclerosis is more common in females, which may influence the reporting distribution of related immunomodulatory agents. Therefore, sex-stratified findings are more appropriately regarded as clinical risk indicators and hypothesis-generating evidence, rather than as direct evidence of biological susceptibility differences.

Several prominent findings were broadly consistent with previously recognized pharmacological or clinical associations. For example, the signals observed for GABAPENTIN, PREGABALIN, CLOZAPINE, BUPROPION, and TRAMADOL were compatible with existing evidence regarding abnormal movements, neurotoxicity, or a reduced seizure threshold. These results therefore mainly strengthen the real-world reporting evidence for established or biologically plausible associations. In contrast, drugs with limited previous evidence, unusually large disproportionality estimates, or occurrence predominantly in one subgroup or database should be considered less well-characterized observations. Such findings may represent potential new safety hypotheses, but they may also be influenced by sparse data, therapeutic context, or reporting bias and require confirmation in independent clinical or population-based studies.

### 4.3 Time-to-onset characteristics and risk management

The TTO analysis showed that the selected signal-associated drugs had Weibull shape parameters below 1, indicating that the corresponding reports were concentrated relatively early after treatment initiation. Although this pattern does not directly represent the time-dependent incidence of these events, it suggests that the early period following treatment initiation, dose escalation, drug switching, changes in concomitant medications, or deterioration of renal function may be a clinically relevant period for heightened awareness.

Among myoclonus-related drugs, the median time to onset was 16.5 days for PREGABALIN and 45.0 days for GABAPENTIN, suggesting that enhanced monitoring is warranted within the first few days to weeks after treatment initiation, particularly in elderly patients, those with renal impairment, patients receiving multiple medications, and those undergoing rapid dose escalation. LAMOTRIGINE showed a longer median time to onset of 214.0 days and a wider distribution range, indicating that lamotrigine-related myoclonus may have a certain degree of delayed onset and heterogeneity. In patients receiving long-term lamotrigine therapy, drug-related myoclonus should still be considered if new-onset myoclonus or abnormal movements occur, even after the early treatment period has passed (Zhou et al., 2022).

Among epileptic seizure-related drugs, the median time to onset was 31.0 days for TRAMADOL and 49.5 days for BUPROPION, suggesting that the early stage to the first several weeks after drug initiation is a key period for monitoring. For drugs that may lower the seizure threshold, clinicians should assess a history of epilepsy, brain injury, alcohol use or withdrawal, metabolic disorders, electrolyte abnormalities, and concomitant medications before treatment initiation, and unnecessary rapid dose escalation or high-dose exposure should be avoided (Wei et al., 2025). INTERFERON BETA 1 A showed a longer median time to onset of 387.0 days, suggesting that neurological events during long-term immunomodulatory therapy may be jointly influenced by underlying disease progression, prolonged drug exposure, and concomitant factors. Therefore, risk management should not be limited to the initial stage of treatment.

The cross-database consistency assessment showed that several drugs reported in FAERS were also represented in CVARD. For myoclonus-related events, CLOZAPINE, GABAPENTIN, BACLOFEN, PREGABALIN, MORPHINE, FENTANYL, SERTRALINE, and PAROXETINE had relatively notable report counts in CVARD. For seizure-related events, corresponding reports were also observed for CLOZAPINE, SERTRALINE, BUPROPION, BACLOFEN, QUETIAPINE, and OLANZAPINE. These overlapping patterns provide evidence of cross-database reporting consistency but do not independently confirm the underlying drug–event associations. Substantial database-specific differences remained and may reflect geographic coverage, drug availability, prescribing practices, approval timelines, population characteristics, coding procedures, reporting behavior, and drug-name standardization. Sparse reporting and database-specific biases may also contribute to unstable or false-positive findings; therefore, both shared and database-specific observations require cautious interpretation.

## 5 Limitations

This study should be interpreted in light of the inherent constraints of spontaneous reporting data. Because FAERS and CVARD do not provide reliable denominators for the number of exposed patients, the observed disproportionality signals reflect reporting associations rather than incidence, absolute risk, relative risk, or confirmed causal relationships. Reporting patterns may also be influenced by underreporting, selective reporting, and heightened attention following regulatory warnings, label changes, published case reports, or increased clinical awareness. Such notoriety or stimulated-reporting effects may exaggerate some associations, whereas highly reported drug–event pairs may reduce the visibility of other potential signals through masking effects. Confounding by indication and channeling bias are also important considerations, because some drugs are preferentially prescribed to patients whose underlying neurological disorders, disease severity, renal impairment, metabolic abnormalities, or complex comorbidities may themselves increase the likelihood of myoclonus or seizure-related events. Although the analysis was restricted to drugs designated as primary suspects, this reporter-assigned role does not constitute a formal causality assessment, particularly in reports involving multiple suspected drugs, concomitant medications, drug interactions, dose changes, or disease progression. In addition, clinical information such as dose, treatment duration, renal and hepatic function, electrolyte levels, neurological history, electroencephalographic findings, imaging results, and concomitant therapies was frequently incomplete, limiting adjustment for potential confounders and the clinical interpretation of individual reports. Variation in MedDRA coding and the absence of independent clinical adjudication may also have resulted in incomplete case identification or phenotype misclassification. The sex-stratified findings should likewise be interpreted cautiously because missing sex information and sparse drug-specific subgroups reduced the feasibility of reliable formal comparisons. Similarly, the time-to-onset analysis included only reports with complete and valid treatment-start and event-onset dates, which may have introduced selection bias. Finally, differences between FAERS and CVARD in geographic coverage, prescribing practices, drug availability, coding procedures, database structure, and reporting behavior limit direct comparability; therefore, cross-database overlap indicates reporting consistency but does not independently confirm causality. Further evaluation using electronic health records, prescription databases, active surveillance systems, and population-based studies is needed to determine the clinical relevance of the identified associations.

## 6 Conclusion

This study characterized the real-world reporting spectrum of myoclonus-related and seizure-related adverse events. Nervous system agents were most frequently represented, while antiinfectives, antineoplastic and immunomodulating agents, metabolic drugs, and cardiovascular drugs were also involved. GABAPENTIN, PREGABALIN, CLOZAPINE, QUETIAPINE, VENLAFAXINE, SERTRALINE, BUPROPION, TRAMADOL, BACLOFEN, and LIDOCAINE were repeatedly identified across different analyses and may represent clinically relevant safety signals warranting further evaluation.

The selected drugs generally showed early-failure TTO patterns, indicating that the corresponding reports were more frequently concentrated during the early period after treatment initiation. However, delayed and widely distributed onset patterns were also observed for some drugs, supporting drug-specific rather than uniform monitoring considerations. Clinical interpretation should integrate the therapeutic indication, baseline neurological condition, renal function, concomitant medications, comorbidity burden, and timing of drug exposure.

Overall, these findings provide hypothesis-generating evidence for recognizing potential medication-associated neurological events. Further population-based and clinically adjudicated studies are required to confirm the associations and determine their actual clinical risks.

## Data Availability

The original contributions presented in the study are included in the article/Supplementary Material, further inquiries can be directed to the corresponding authors.
