# Supplementary material for: Sex-specific pharmacovigilance signals and time-to-onset patterns of drug-related myoclonus and epileptic seizures: a real-world pharmacovigilance analysis
Source: Front Pharmacol. 2026 Aug 25;17:1907007. doi: 10.3389/fphar.2026.1907007 (PMC13550190; doi:10.3389/fphar.2026.1907007)
Supplement: Supplementary file 1 [file Supplementaryfile1.docx]

Supplementary Material 1 Two-by-two contingency table for disproportionality analyses.

| Drugs | Target AEs | Other AEs |
| --- | --- | --- |
| Target drugs | a | b |
| Other drugs | c | d |
| Total | a+c | b+d |
| Algorithms | Equation | Criteria |
| ROR | ROR=ad/b/c | lower limit of 95% CI>1, N≥3 |
|  | 95%CI=e^ln(ROR)±1.96(1/a+1/b+1/c+1/d)^0.5^ |  |

Abbreviation: AEs, adverse events; a, number of reports containing both the target drug and target adverse drug reaction; b, number of reports containing other adverse drug reaction of the target drug; c, number of reports containing the target adverse drug reaction of other drugs; d, number of reports containing other drugs and other adverse drug reactions.
