# Supplementary material for: Sex-specific pharmacovigilance signals and time-to-onset patterns of drug-related myoclonus and epileptic seizures: a real-world pharmacovigilance analysis
Source: Front Pharmacol. 2026 Aug 25;17:1907007. doi: 10.3389/fphar.2026.1907007 (PMC13550190; doi:10.3389/fphar.2026.1907007)
Supplement: Supplementary file 2 [file Supplementaryfile2.docx]

**Supplementary Table S1. MedDRA Preferred Terms used to identify myoclonus-related and seizure-related events**

**Table S1A. Preferred Terms used to identify myoclonus-related events**

| No. | Descriptive category | MedDRA Preferred Term |
| --- | --- | --- |
| 1 | Core myoclonus phenotype | MYOCLONUS |
| 2 | Core myoclonus phenotype | OPSOCLONUS MYOCLONUS |
| 3 | Core myoclonus phenotype | POST-ANOXIC MYOCLONUS |
| 4 | Core myoclonus phenotype | EYELID MYOCLONUS |
| 5 | Myoclonus-associated movement disorder | MYOCLONIC DYSTONIA |
| 6 | Myokymia-related phenotype | MYOKYMIA |
| 7 | Myokymia-related phenotype | SUPERIOR OBLIQUE MYOKYMIA |
| 8 | Myoclonic epilepsy or syndrome | MYOCLONIC EPILEPSY |
| 9 | Myoclonic epilepsy or syndrome | LAFORA'S MYOCLONIC EPILEPSY |
| 10 | Myoclonic epilepsy or syndrome | BALTIC MYOCLONIC EPILEPSY |
| 11 | Myoclonic epilepsy or syndrome | JUVENILE MYOCLONIC EPILEPSY |
| 12 | Myoclonic epilepsy or syndrome | MYOCLONIC EPILEPSY AND RAGGED-RED FIBRES |
| 13 | Myoclonic epilepsy or syndrome | SEVERE MYOCLONIC EPILEPSY OF INFANCY |
| 14 | Myoclonic epilepsy or syndrome | EPILEPSY WITH MYOCLONIC-ATONIC SEIZURES |
| 15 | Myoclonic epilepsy or syndrome | PROGRESSIVE MYOCLONIC EPILEPSY |

**Table S1B. Preferred Terms used to identify seizure-related events**

| No. | Descriptive category | MedDRA Preferred Term |
| --- | --- | --- |
| 1 | Metabolic or acute symptomatic seizure | HYPOGLYCAEMIC SEIZURE |
| 2 | Metabolic or acute symptomatic seizure | HYPERGLYCAEMIC SEIZURE |
| 3 | Metabolic or acute symptomatic seizure | HYPOCALCAEMIC SEIZURE |
| 4 | Metabolic or acute symptomatic seizure | HYPONATRAEMIC SEIZURE |
| 5 | Anoxic or structural seizure | SEIZURE ANOXIC |
| 6 | Anoxic or structural seizure | POST STROKE SEIZURE |
| 7 | Focal or subtype-specific seizure | PARTIAL SEIZURES |
| 8 | Focal or subtype-specific seizure | AUTONOMIC SEIZURE |
| 9 | Focal or subtype-specific seizure | GELASTIC SEIZURE |
| 10 | Focal or subtype-specific seizure | TARDIVE SEIZURE |
| 11 | Focal or subtype-specific seizure | ACUTE ENCEPHALITIS WITH REFRACTORY, REPETITIVE PARTIAL SEIZURES |
| 12 | Neonatal seizure | NEONATAL EPILEPTIC SEIZURE |
| 13 | Generalized or convulsive seizure | GENERALISED TONIC-CLONIC SEIZURE |
| 14 | Generalized or convulsive seizure | GRAND MAL CONVULSION |
| 15 | Seizure-associated clinical syndrome | ICTAL BRADYCARDIA SYNDROME |
| 16 | Nonepileptic seizure-like event | PSYCHOGENIC SEIZURE |

**Abbreviations:** MedDRA, Medical Dictionary for Regulatory Activities; PT, Preferred Term. Myoclonus-related and seizure-related events were identified using two prespecified sets of PTs from MedDRA version 28.0. The terms shown in this table are reproduced exactly as used in the data-extraction procedure. The descriptive categories were introduced solely to improve the presentation of the search strategy and were not used as separate analytical outcomes. A case was considered eligible when at least one corresponding PT was recorded. When multiple eligible PTs belonging to the same outcome category were reported within one case, the case was counted once at the case–outcome level. Cases meeting the definitions of both outcome categories were permitted to contribute to both analyses.
